# Comparative activity of newer β-lactam/β-lactamase inhibitor combinations against Pseudomonas aeruginosa from patients hospitalized with pneumonia in European medical centers in 2020

**DOI:** 10.1007/s10096-021-04363-7

**Published:** 2021-10-16

**Authors:** Helio S. Sader, Cecilia G. Carvalhaes, Dee Shortridge, Mariana Castanheira

**Affiliations:** JMI Laboratories, 345 Beaver Kreek Centre, Suite A, North Liberty, IA 52317 USA

**Keywords:** *Pseudomonas aeruginosa*, Ceftazidime-avibactam, Ceftolozane-tazobactam, Imipenem-relebactam, Meropenem-vaborbactam

## Abstract

*Pseudomonas aeruginosa* isolates were consecutively collected from patients with pneumonia in 29 medical centers in 2020 and susceptibility tested by broth microdilution method. Ceftazidime-avibactam (95.5% susceptible), imipenem-relebactam (94.3% susceptible), and ceftolozane-tazobactam (93.3% susceptible) were the most active compounds after colistin (99.5% susceptible). Susceptibility rates for the β-lactam/β-lactamase inhibitor combinations (BL/BLIs) varied against isolates resistant to piperacillin-tazobactam, meropenem, imipenem, and/or ceftazidime. Ceftazidime-avibactam was the most active BL/BLI against resistant subsets from Western Europe, whereas imipenem-relebactam was slightly more active than other BL/BLIs against resistant subsets from Eastern Europe. Susceptibility rates were markedly lower in Eastern Europe than Western Europe.

## Introduction

The initial antimicrobial therapy of patients with pneumonia is frequently empirical, and the most appropriate empirical regimen is determined mainly by understanding causative pathogens and the antimicrobial susceptibility of these organisms. Moreover, the implementation of timely and effective antimicrobial therapy is critical to decrease complications and mortality [1–3].

*Pseudomonas aeruginosa* is one of the most common organisms isolated from respiratory samples of patients with pneumonia in European medical centers [3, 4] and represents a serious therapeutic challenge because it exhibits intrinsically decreased susceptibility to a range of antimicrobials and possesses a great ability to acquire and/or develop a diversity of resistant traits that can affect one or multiple antimicrobial agents [5, 6]. *P. aeruginosa* carries an inducible AmpC cephalosporinase, which can cause resistance to anti-pseudomonal cephalosporins and piperacillin-tazobactam when its production is significantly increased. Furthermore, upregulation of MexA-MexB-OprM and the loss of OprD are considered the most prevalent mechanisms of carbapenem resistance in *P. aeruginosa*; these mechanisms are usually associated with AmpC hyperproduction [7]. It is also important to note that infections caused by multidrug-resistant (MDR) *P. aeruginosa* strains and a delay in appropriate antimicrobial therapy for serious *P. aeruginosa* infections are associated with longer hospital stays and increased mortality [8].

The most prominent group of new antimicrobial agents with broad spectrum activity is the β-lactam/β-lactamase inhibitor combinations (BL/BLI). Four such combinations have been approved in recent years: ceftazidime-avibactam, ceftolozane-tazobactam, meropenem-vaborbactam, and imipenem-relebactam. Many others are currently in different stages of development and approval [9]. In this study, we evaluated the in vitro activity of these 4 most recently approved BL/BLIs against *P. aeruginosa* isolates recovered from respiratory samples of patients hospitalized with pneumonia in European hospitals in 2020.

## Materials and methods

Bacterial isolates were collected via the SENTRY Antimicrobial Surveillance Program and sent to JMI Laboratories (North Liberty, IA, USA) for susceptibility testing [10]. Each participating center was asked to collect 100 consecutive bacterial isolates from respiratory specimens determined to be significant by local criteria as the reported probable cause of pneumonia. Qualified sputum samples and isolates from invasive sampling, such as transtracheal aspiration, bronchoalveolar lavage, and protected brush samples, were accepted.

A total of 2,793 bacterial isolates were collected in 2020, including the 583 *P. aeruginosa* evaluated in this study. Isolates were collected from 29 medical centers located in Western Europe (W-EU; *n* = 401; 21 centers in 10 countries [Belgium, France, Germany, Ireland, Italy, Portugal, Spain, Sweden, Switzerland, and the UK]) and the Eastern European and Mediterranean region (E-EU; *n* = 182; 8 centers in 8 countries [Czech Republic, Greece, Hungary, Israel, Poland, Romania, Slovenia, and Turkey]). Species identification was confirmed by using standard biochemical tests and/or a MALDI Biotyper (Bruker Daltonics, Billerica, MA, USA), when necessary.

All isolates were susceptibility tested using the reference broth microdilution method at a monitoring laboratory (JMI Laboratories, North Liberty, IA, USA) as described by the CLSI [11]. Ceftazidime-avibactam, ceftolozane-tazobactam, imipenem-relebactam, and piperacillin-tazobactam were tested with the β-lactamase inhibitor at fixed concentration of 4 mg/L; meropenem-vaborbactam was tested with vaborbactam at fixed concentration of 8 mg/L [11, 12]. MIC results were interpreted according to EUCAST breakpoint criteria [13].

## Results

Overall, ceftazidime-avibactam (MIC_50/90_, 2/8 mg/L; 95.5% susceptible), imipenem-relebactam (MIC_50/90_, 0.25/2 mg/L; 94.3% susceptible), and ceftolozane-tazobactam (MIC_50/90_, 0.5/2 mg/L; 93.3% susceptible) were the most active compounds against *P. aeruginosa* isolates after colistin (99.5% susceptible; Table 1). All four newer BL/BLIs were active against > 90% of *P. aeruginosa* isolates from W-EU. According to current EUCAST breakpoints criteria, ceftazidime-avibactam (MIC_50/90_, 2/4 mg/L) showed the highest susceptibility rate (97.2%) against isolates from W-EU, followed by imipenem-relebactam (MIC_50/90_, 0.25/1 mg/L; 94.5% susceptible), ceftolozane-tazobactam (MIC_50/90_, 0.5/2 mg/L; 94.3% susceptible), and meropenem-vaborbactam (MIC_50/90_, 0.5/8 mg/L; 91.0% susceptible; Table 1). It is important to note that the higher susceptibility rates of meropenem-vaborbactam in comparison with meropenem is a result of the different breakpoints applied to the 2 compounds (≤ 2 mg/L for meropenem and ≤ 8 mg/L for meropenem/vaborbactam) rather than a substantial improvement in activity, as evidenced by the near-identical MIC_50/90_ and percentage of resistance values (Table 1).

**Table 1:** Antimicrobial activity of ceftazidime-avibactam, ceftolozane-tazobactam, meropenem-vaborbactam, imipenem-relebactam, and comparators against *P. aeruginosa* recovered from patients with pneumonia in European hospitals in 2020

Antimicrobial agent	W-EU (401 isolates)						E-EU (182 isolates)						All isolates (583)				
	MIC_50_	MIC_90_	%*S*^a^	%*I*^a^	%*R*^a^	MIC_50_		MIC_90_	%*S*^a^	%*I*^a^	%*R*^a^	MIC_50_		MIC_90_	%*S*	%I	%R
Ceftazidime-avibactam	2	4	97.2		2.8	2		8	91.8		8.2	2		8	95.5		4.5
Ceftolozane-tazobactam	0.5	2	94.3		5.7	0.5		4	91.2		8.8	0.5		2	93.3		6.7
Imipenem-relebactam	0.25	1	94.5		5.5	0.25		2	94.0		6.0	0.25		2	94.3		5.7
Meropenem-vaborbactam	0.5	8	91.0		9.0	0.5		16	83.5		16.5	0.5		16	88.7		11.3
Piperacillin-tazobactam	4	128	^b^	74.6	25.4	4		128	^b^	72.0	28.0	4		128	^b^	73.8	26.2
Ceftazidime	2	32	^b^	77.6	22.4	2		32	^b^	78.6	21.4	2		32	^b^	77.9	22.1
Cefepime	2	16	^b^	81.5	18.5	4		32	^b^	83.0	17.0	2		16	^b^	82.0	18.0
Meropenem^c^	0.5	8	75.8	14.5	9.7	1		16	65.4	17.6	17.0	0.5		16	72.6	15.4	12.0
Imipenem	1	> 8	^b^	76.1	23.9	1		> 8	^b^	67.6	32.4	1		> 8	^b^	73.4	26.6
Tobramycin	0.5	2	91.8 ^d^		8.2	1		> 16	84.6 ^d^		15.4	0.5		4	89.5 ^d^		10.5
Amikacin	4	8	94.0 ^d^		6.0	4		32	87.9 ^d^		12.1	4		16	92.1 ^d^		7.9
Levofloxacin	0.5	8	^b^	68.8	31.2	1		32	^b^	62.1	37.9	0.5		16	^b^	66.7	33.3
Colistin	0.5	1	99.7		0.3	1		1	98.9		1.1	1		1	99.5		0.5

^a^Percentages considered susceptible, intermediate, and resistant follow EUCAST criteria [13]

^b^An arbitrary susceptible breakpoint of ≤ 0.001 mg/L has been published by EUCAST indicating that susceptible should not be reported for this organism-agent combination and intermediate should be interpreted as susceptible increased exposure

^c^Non-meningitis breakpoints were applied

^d^For infections originating from the urinary tract. For systemic infections, aminoglycosides must be used in combination with other active therapy [13]

In general, susceptibility rates were slightly lower among isolates from E-EU, and the highest susceptibility rate was shown by imipenem-relebactam (MIC_50/90_, 0.25/2 mg/L; 94.0% susceptible), followed by ceftazidime-avibactam (MIC_50/90_, 2/8 mg/L; 91.8% susceptible), ceftolozane-tazobactam (MIC_50/90_, 0.5/4 mg/L; 91.2% susceptible), and meropenem-vaborbactam (MIC_50/90_, 0.5/16 mg/L; 83.5% susceptible; Table 1). The most active comparator agents were colistin (99.7% and 98.9% susceptible in W-EU and E-EU, respectively), amikacin (94.0% and 87.9% susceptible in W-EU and E-EU, respectively), and tobramycin (91.8% and 84.6% susceptible in W-EU and E-EU, respectively; Table 1).

Susceptibility rates for the BL/BLIs varied more broadly against isolates resistant to piperacillin-tazobactam, meropenem, imipenem, or ceftazidime (Table 2). Ceftazidime-avibactam was the most active BL/BLI against these resistant subsets from W-EU, with susceptibility rates ranging from 92.6% when tested against imipenem-resistant isolates to 87.8% against ceftazidime-resistant strains. Imipenem-relebactam was slightly more active than the other BL/BLIs against resistant subsets from E-EU. Imipenem-relebactam susceptibility rates against resistant subsets from E-EU ranged from 81.4% against imipenem-resistant isolates to 64.5% against meropenem-resistant strains (Table 2). Moreover, ceftazidime-avibactam retained activity against 81.2% of W-EU isolates resistant to piperacillin-tazobactam, imipenem, meropenem, and ceftazidime, whereas ceftolozane-tazobactam, imipenem-relebactam, and meropenem-vaborbactam were active against 59.0%, 53.8%, and 7.7% of these isolates, respectively (Table 2). Imipenem-relebactam was the most active agent against E-EU isolates resistant to these 4 β-lactam compounds, inhibiting 64.5% at the EUCAST susceptible breakpoint of ≤ 2 mg/L (Table 2).

**Table 2:** Antimicrobial activity of ceftazidime-avibactam, ceftolozane-tazobactam, meropenem-vaborbactam, imipenem-relebactam, and comparators against resistant subsets of *P. aeruginosa* isolates from patients with pneumonia in European hospitals in 2020

Resistant phenotype	% Susceptible per EUCAST (no. of isolates)		
	W-EU	E-EU	All isolates
Piperacillin-tazobactam-resistant (MIC > 16 mg/L)	(102)	(51)	(153)
Ceftazidime-avibactam	91.2	70.6	84.3
Ceftolozane-tazobactam	78.4	70.0	75.7
Imipenem-relebactam	83.3	78.4	81.7
Meropenem-vaborbactam	69.6	49.0	62.7
Meropenem-resistant (MIC > 8 mg/L)	(39)	(31)	(70)
Ceftazidime-avibactam	81.6	54.8	69.9
Ceftolozane-tazobactam	59.0	54.8	57.1
Imipenem-relebactam	53.8	64.5	58.6
Meropenem-vaborbactam	7.7	3.2	5.7
Imipenem-resistant (MIC > 4 mg/L)	(96)	(59)	(155)
Ceftazidime-avibactam	92.6	78.0	87.0
Ceftolozane-tazobactam	84.4	78.0	81.9
Imipenem-relebactam	77.1	81.4	78.7
Meropenem-vaborbactam	63.5	52.5	59.4
Ceftazidime-resistant (MIC > 8 mg/L)	(90)	(39)	(129)
Ceftazidime-avibactam	87.8	61.5	79.8
Ceftolozane-tazobactam	75.6	61.5	71.3
Imipenem-relebactam	81.1	74.4	79.1
Meropenem-vaborbactam	72.2	46.2	64.3
β-lactam-resistant^a^	(16)	(9)	(25)
Ceftazidime-avibactam	81.2	33.3	64.0
Ceftolozane-tazobactam	43.8	55.6	48.0
Imipenem-relebactam	43.8	55.6	48.0
Meropenem-vaborbactam	6.2	0.0	4.0

^a^Isolates resistant to piperacillin-tazobactam (MIC > 16 mg/L), meropenem (MIC > 8 mg/L), imipenem(MIC > 4 mg/L), and ceftazidime (MIC > 8 mg/L) per EUCAST criteria [13]

Abbreviations: *W-EU* Western Europe, *E-EU* Eastern Europe

## Discussion

The treatment of *P. aeruginosa* pneumonia represents a great challenge for physicians. *P. aeruginosa* is an organism for which very few therapeutic options are clinically available, and resistance to tractional anti-pseudomonal β-lactams, such as piperacillin-tazobactam, ceftazidime, cefepime, imipenem, and meropenem, is elevated in many geographic regions. Moreover, non-β-lactam agents that are active against *P. aeruginosa*, such as aminoglycosides, colistin, and fosfomycin, are limited in their efficacy, safety profile, and/or by the emergence of resistance [2, 7, 8].

Novel BL/BLIs represent valuable new therapeutic options for *P. aeruginosa* infections, for which limited treatment options were available [9]. In the present study, we evaluated the antimicrobial susceptibility of contemporary (2020) isolates of *P. aeruginosa* recovered from respiratory samples of patients with pneumonia. Our results complement the results of other surveillance programs by providing comparative results for the four BL/BLIs most recently approved for the treatment of *P. aeruginosa* pneumonia in Europe [14–17]. Other surveillance networks, like the European Antimicrobial Resistance Surveillance Network (EARS-NET), evaluates the antimicrobial susceptibility of *P. aeruginosa* in many European countries and publishes valuable data periodically, but the activities of these new BL/BLIs are not evaluated in EARS-NET or other large surveillance programs [18, 19].

The results of this investigation showed that, besides colistin, the new BL/BLIs were the most active compounds against *P. aeruginosa*, with susceptibility rates similar to the aminoglycosides tobramycin and amikacin. Our results also showed that these new BL/BLIs, especially ceftazidime-avibactam, imipenem-relebactam, and ceftolozane-tazobactam, retained good activity against *P. aeruginosa* isolates resistant to β-lactams currently used to treat *P. aeruginosa* infections.

Another interesting finding was the regional variation of the activity of these BL/BLIs within Europe. Ceftazidime-avibactam was the most active agent against isolates from W-EU with 97.2% susceptibility, followed by imipenem-relebactam (94.5% susceptible) and ceftolozane-tazobactam (94.3% susceptible). Imipenem-relebactam was the most active BL/BLI against isolates from E-EU (94.0% susceptible), followed by ceftazidime-avibactam (91.8% susceptible) and ceftolozane-tazobactam (91.2% susceptible; Table 1). Regional differences on the activities of these BL/BLIs reflect the variety of resistance mechanisms expressed by *P. aeruginosa* and illustrate how these mechanisms may have different impacts on each of these compounds. Mechanisms of resistance to these new BL/BLIs are usually very complex and caused by the presence and interaction of multiple mutation-driven resistance mechanisms [20, 21]. Therefore, the activity of these compounds, and especially the rates of cross-resistance between them, may vary widely depending on selective pressure due to previous antibiotic usage.

The limitations of the study should be considered when interpreting the results and conclusions. First, the criteria used to categorize a bacterial isolate as clinically significant were not defined in the study protocol and were based on local algorithms. Second, due to the lack of clinical information available, this study could not exclude the possibility that some organisms were colonizers. Third, a limited number of isolates and/or medical centers were surveyed in some European countries; thus, the results presented here may not represent the overall picture from those European regions.

In conclusion, the recently approved BL/BLIs demonstrated potent activity and broad coverage against *P. aeruginosa* isolated from patients with pneumonia in European medical centers. Based on the current EUCAST breakpoints, ceftazidime-avibactam, ceftolozane-tazobactam, and imipenem-relebactam showed similar overall coverage (% susceptible) against *P. aeruginosa*, while susceptibility rates were lower for meropenem-vaborbactam, especially against resistant subsets. Moreover, susceptibility rates were markedly lower in E-EU compared to W-EU.
